# A three-dimensional tissue-engineered rostral migratory stream as an *in vitro* platform for subventricular zone-derived cell migration

**DOI:** 10.3389/fbioe.2024.1410717

**Published:** 2024-06-12

**Authors:** Erin M. Purvis, Andrés D. Garcia-Epelboim, Elizabeth N. Krizman, John C. O’Donnell, D. Kacy Cullen

**Affiliations:** ^1^ Center for Brain Injury and Repair, Department of Neurosurgery, Perelman School of Medicine, University of Pennsylvania, Philadelphia, PA, United States; ^2^ Center for Neurotrauma, Neurodegeneration and Restoration, Corporal Michael J. Crescenz Veterans Affairs Medical Center, Philadelphia, PA, United States; ^3^ Department of Neuroscience, Perelman School of Medicine, University of Pennsylvania, Philadelphia, PA, United States; ^4^ Department of Physics and Astronomy, School of Arts and Sciences, University of Pennsylvania, Philadelphia, PA, United States; ^5^ Department of Bioengineering, School of Engineering and Applied Science, University of Pennsylvania, Philadelphia, PA, United States

**Keywords:** rostral migratory stream, subventricular zone, astrocyte, tissue engineering, cell morphology, cell migration, neuroblast migration

## Abstract

In the brains of most adult mammals, neural precursor cells (NPCs) from the subventricular zone (SVZ) migrate through the rostral migratory stream (RMS) to replace olfactory bulb interneurons. Following brain injury, published studies have shown that NPCs can divert from the SVZ-RMS-OB route and migrate toward injured brain regions, but the quantity of arriving cells, the lack of survival and terminal differentiation of neuroblasts into neurons, and their limited capacity to re-connect into circuitry are insufficient to promote functional recovery in the absence of therapeutic intervention. Our lab has fabricated a biomimetic tissue-engineered rostral migratory stream (TE-RMS) that replicates some notable structural and functional components of the endogenous rat RMS. Based on the design attributes for the TE-RMS platform, it may serve as a regenerative medicine strategy to facilitate sustained neuronal replacement into an injured brain region or an *in vitro* tool to investigate cell-cell communication and neuroblast migration. Previous work has demonstrated that the TE-RMS replicates the basic structure, unique nuclear shape, cytoskeletal arrangement, and surface protein expression of the endogenous rat RMS. Here, we developed an enhanced TE-RMS fabrication method in hydrogel microchannels that allowed more robust and high-throughput TE-RMS assembly. We report unique astrocyte behavior, including astrocyte bundling into the TE-RMS, the presence of multiple TE-RMS bundles, and observations of discontinuities in TE-RMS bundles, when microtissues are fabricated in agarose microchannels containing different critical curved or straight geometric features. We also demonstrate that we can harvest NPCs from the SVZ of adult rat brains and that EGFP+ cells migrate in chain formation from SVZ neurospheres through the TE-RMS *in vitro*. Overall, the TE-RMS can be utilized as an *in vitro* platform to investigate the pivotal cell-cell signaling mechanisms underlying the synergy of molecular cues involved in immature neuronal migration and differentiation.

## Introduction

Neurogenesis occurs in the subventricular zone (SVZ) of most adult mammals (Zhao et al., 2008; Ming and Song, 2011). Neural precursor cells (NPCs) born in the SVZ migrate through the rostral migratory stream (RMS) to the olfactory bulb (OB), where they integrate into existing circuitry as functional OB interneurons (Lledo et al., 2008; Brill et al., 2009; Lazarini and Lledo, 2011; Lim and Alvarez-Buylla, 2016). SVZ neurogenesis is upregulated following brain injury (Tang et al., 2009; Chang et al., 2016; Lu et al., 2017). Neuroblasts are guided by chemo-attractive factors to divert from their regular migration route along the RMS and migrate toward injured brain regions where they can mature into functional, phenotypically relevant neurons and integrate into existing circuitry (Hou et al., 2008; Kaneko et al., 2018; Wang et al., 2020). Functional recovery following injury can be achieved by experimentally enhancing the redirection of neuroblasts from the SVZ to injured brain regions (Ohab et al., 2006; Kolb et al., 2007; Schabitz et al., 2007; Petraglia et al., 2010; Popa-Wagner et al., 2010; Erlandsson et al., 2011; Yu et al., 2013; Clark et al., 2016; Gundelach and Koch, 2018; Jinnou et al., 2018; Kaneko et al., 2018; Wang et al., 2020). We and others have previously reported on the variety of tissue engineering and biomaterial strategies that have been created to augment the redirection of SVZ neuroblasts into injured brain regions (Pettikiriarachchi et al., 2010; Oliveira et al., 2018; Purvis et al., 2020). Our laboratory has developed a biomimetic “living scaffold,” which is a tissue engineering strategy consisting of living cells in a defined anisotropic three-dimensional structure, that replicates some notable structural and functional components of the glial tube of the RMS and thereby provides sustained cell-cell cues (Winter et al., 2016; Katiyar et al., 2018; O’Donnell et al., 2018; O’Donnell et al., 2021; Purvis et al., 2022). This tissue-engineered rostral migratory stream (TE-RMS) is designed to facilitate stable, sustained neuroblast delivery into neuron-deficient brain regions following injury. By replicating the endogenous RMS, which is an intrinsic neural mechanism for endogenous neuronal replacement, we believe this will create the most effective biologically engineered migration path that may lead to continual, robust neuronal replacement. In addition to its potential as an emerging regenerative medicine strategy for neuronal replacement following focal brain injury, the TE-RMS is also a powerful tool to unlock previously undiscovered mechanisms of cell-cell communication and neuroblast migration.

**FIGURE 1:**
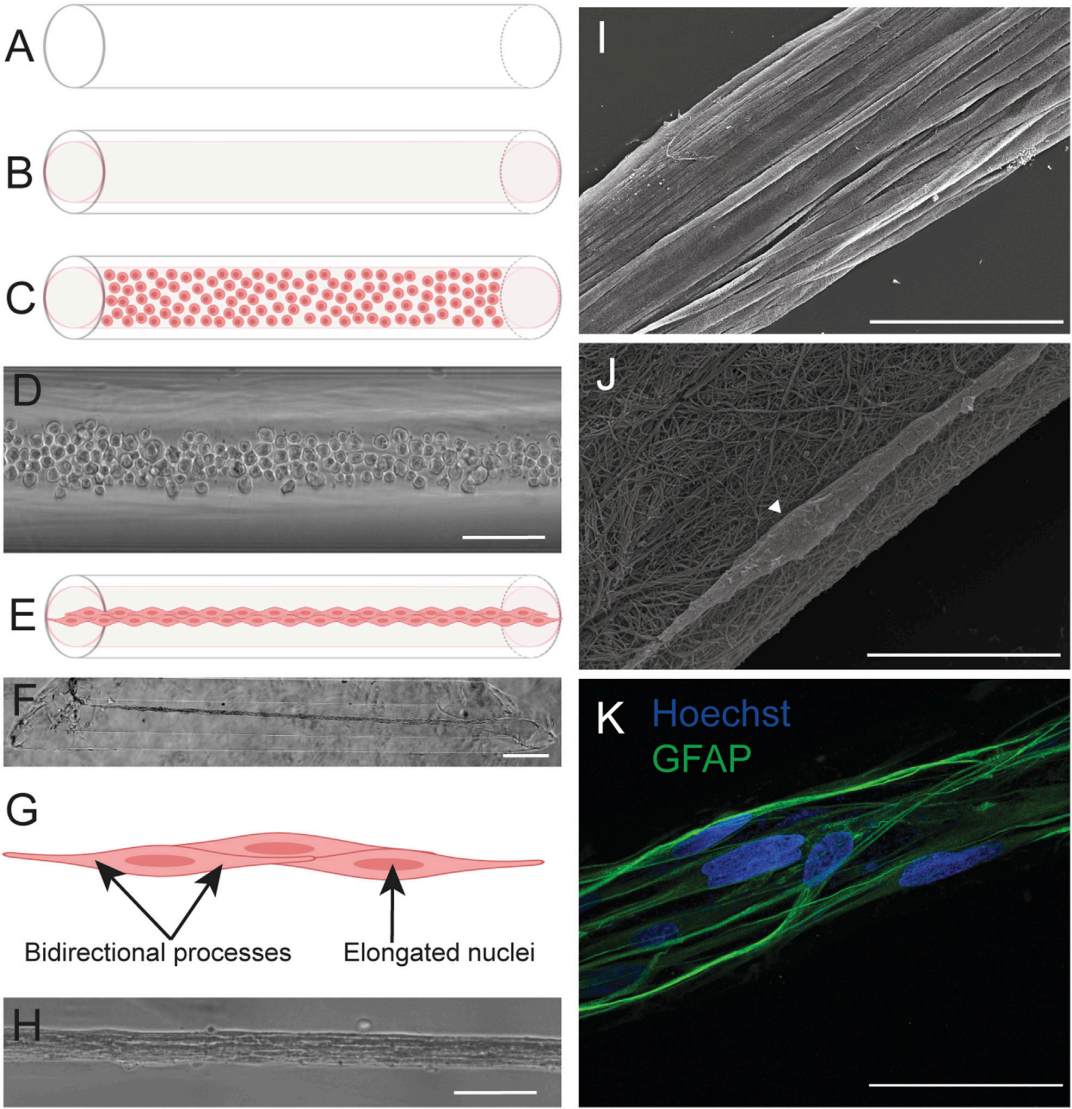
TE-RMS fabrication in hydrogel microcolumns. Our previous work reported the fabrication of TE-RMSs in hydrogel microcolumns. Agarose columns **(A)** are cut at an angle and seeded with rat tail type 1 collagen extracellular matrix **(B)**. Following complete collagen polymerization, columns are seeded with a concentrated solution of living astrocytes **(C,D)**. Astrocytes self-assemble into the TE-RMS in a period of 8–24 h **(E,F)**. TE-RMS astrocytes have elongated nuclei and bidirectional processes that run parallel to the long nuclear axes **(G,H)**. Scanning electron microscopy **(I,J)** and high magnification fluorescence microscopy **(K)** reveal the detailed structure of the TE-RMS as a tight bundle of longitudinally aligned astrocytes coated in a fine meshwork of collagen. Scale bars: 200 microns **(D,H)**, 500 microns **(F)**, 50 microns **(I,K)**, 10 microns **(J)**. Panels adapted from Purvis et al., 2022.

Previous work details the fabrication of the TE-RMS in hydrogel microcolumns (Winter et al., 2016; Katiyar et al., 2018; O’Donnell et al., 2021; Purvis et al., 2022) (Figure 1). TE-RMSs are fabricated in 3% agarose microcolumns with a 300-micron inner diameter. Previous studies have shown these column conditions to be the most optimal to induce TE-RMS formation (Winter et al., 2016). To fabricate TE-RMSs, these hydrogel microcolumns (Figure 1A) are seeded with collagen extracellular matrix (ECM; Figure 1B), then following complete collagen polymerization, columns are seeded with a dense suspension (1 million cells/mL) of astrocytes (Figures 1C,D). In a period of 8–24 h, astrocytes extend their processes longitudinally within the lumen of the hydrogel microcolumn, pull the polymerized collagen off the inner surface of the hydrogel, and self-assemble into aligned bundles of astrocytes that tether to either end of the column (Figures 1E–H). The TE-RMS is thus a rope-like bundle of bidirectional, aligned astrocytes coated in a fine meshwork of collagen (Figures 1I–K). We have previously shown that the TE-RMS fabricated from either rat primary astrocytes or human gingiva mesenchymal stem cell-derived astrocytes mimics the basic structure and key surface protein expression of the endogenous rat RMS (O’Donnell et al., 2021). Additionally, the TE-RMS successfully redirects migration of immature rat cortical neurons *in vitro* and can facilitate the migration of endogenous RMS neuroblasts *in vivo* (O’Donnell et al., 2021). Our recent work also reported on the unique nuclear and cytoskeletal architecture of astrocytes in the TE-RMS, demonstrating profound nuclear elongation and intermediate filament rearrangement of astrocytes in the TE-RMS compared to 2D planar astrocytes (Purvis et al., 2022) (Figure 1G). Further, this distinctive TE-RMS astrocyte architecture mimics that of astrocytes in the endogenous rat RMS but is different from non-RMS astrocytes (Purvis et al., 2022). However, a limiting factor of these previously reported studies was the relatively low-throughput methodology for fabricating the TE-RMS, where each microcolumn–typically presenting a lumen of only 300 microns in diameter–was meticulously seeded with ECM and cells. Thus, we sought to design a more high-throughput TE-RMS fabrication method that would be more sustainable for future utilization of this technology, opening the door to expanded investigations that require large quantities of TE-RMSs.

Accordingly, the current study examines *in vitro* applications of the TE-RMS as a tool to investigate mechanisms of cell-cell communication and neuroblast migration. We describe novel TE-RMS fabrication methods in hydrogel microchannels as a replacement to microcolumns, creating a higher-throughout fabrication procedure that allows for the production of a greater quantity of TE-RMSs in a more rapid fashion. Here, we systematically established the effects of four different microchannel geometries on astrocyte behavior and self-assembly into the TE-RMS. We then show that we can harvest NPCs from the SVZ of EGFP+ adult rat brains and culture these cells as neurospheres. Following loading of SVZ-derived neurospheres to the end of the TE-RMS, EGFP+ cells that are purported to be neuroblasts migrate *in vitro* along TE-RMSs generated using the new fabrication methodology. Notably, this is our first report demonstrating the efficiency of the TE-RMS in facilitating chain migration of SVZ-derived cells *in vitro*. These analyses demonstrate the capability of the TE-RMS to serve as an *in vitro* platform for the investigation of the various cell-cell signaling mechanisms and molecular cues involved in cell migration and maturation.

## Materials and methods

### Isolation and culture of astrocytes

All procedures described in this manuscript adhere to the National Institutes of Health Guide for the Care and Use of Laboratory Animals and were approved by the Institutional Animal Care and Use Committee at the University of Pennsylvania. Primary cortical astrocytes were harvested from postnatal day 0–1 Sprague-Dawley rat pups (male or female) (McCarthy and de Vellis, 1980). Astrocytes were isolated from non-transgenic rats. Following dissociation [described previously in (Katiyar et al., 2018)], astrocytes were cultured in Dulbecco’s Modified Eagle Medium F12 (DMEM/F12; Gibco #11330032) supplemented with 10% Fetal Bovine Serum (Sigma #F0926) and 1% Penicillin-Streptomycin (Gibco #15140122) antibiotics in a cell culture incubator maintained at 37°C and 5% CO_2_. Astrocyte flasks were passaged with trypsin-EDTA (Gibco #25200056) at 80% confluency to maintain astrocyte cell lines. Astrocytes between passages 4–8 were used for TE-RMS fabrication (McCarthy and de Vellis, 1980; O’Donnell et al., 2021; Purvis et al., 2022).

### Isolation and culture of subventricular zone cells

SVZ cells were harvested from adult (at least two-month-old) transgenic rats (male or female) that expressed EGFP. Three different rat strains that constitutively expressed EGFP [SD-Tg(UBC-EGFP)2BalRrrc (heterozygous), LEW-Tg(CAG-EGFP)YsRrrc (hemizygous), F344-Tg(UBC-EGFP)F455Rrrc (homozygous)] were utilized for harvesting SVZ cells. Cells from Sprague-Dawley strain SD-Tg(UBC-EGFP)2BalRrrc were utilized for all migration studies. Procedures described here were adopted from (Vazey et al., 2006; Chen et al., 2007). Following subject overdose with CO_2_ and subsequent decapitation, brains were extracted from the skull and placed in cold Leibovitz’s L-15 media (Gibco #11415064). The brain was placed ventral side up and a coronal cut was made at the optic chiasm. The SVZ, including the ependyma and subependyma, were dissected from the striatal side of the lateral ventricles. Tissue was minced and digested in media containing a base of L-15 media supplemented with 2.5 U/mL papain (Sigma #P4762), 10 U/mL DNase (Roche #10104159001), and 100 U/mL Penicillin-Streptomycin (Gibco #15140122) for 40 min at 37°C. The tube was agitated every few minutes during the digestion period. The solution was then triturated and centrifuged for 3 min at 201 g. Following removal of supernatant, tissue was re-suspended in media containing a base of DMEM/F12 media (Gibco #11330032) supplemented with 2% B27, 10 U/mL DNase, 3 mg/mL glucose (Sigma #G7528), 100 U/mL Penicillin-Streptomycin (Gibco #15140122) for 15 min at 37°C. The tube was agitated every few minutes during this period. The solution was then triturated and centrifuged for 3 min at 201 g. Supernatant was removed and replaced with warm DMEM/F12 media (Gibco #11330032). Solution was triturated and passed through a 40-micron cell strainer. The resulting solution was centrifuged for 3 min at 201 g. Supernatant was removed and cells were suspended in SVZ culture media containing a base of Neurobasal media (Gibco #21103049) supplemented with 2% B27 (Invitrogen #12587010), 3 mg/mL glucose (Sigma #G7528), 2 mM L-glutamine (Gibco #35050061), 100 U/mL Penicillin-Streptomycin (Gibco #15140122), 20 ng/mL human recombinant epidermal growth factor (EGF) (Millipore # GF144), 20 ng/mL human recombinant fibroblast growth factor (FGF) (Millipore #GF003-AF), and 2ug/mL heparin (Stem Cell #07980) (Supplementary Table S1). Cells were plated in a 96-well plate at a density of 200,000 cells/mL. Cells were fed every 2–3 days by replacing half of the media. SVZ neurospheres were large enough to visually transport at DIV7, but not so large that the inside of the neurospheres had started to become necrotic. Thus, DIV7 neurospheres were utilized for all experiments herein.

### Fabrication of molds and hydrogel microchannels

**FIGURE 2:**
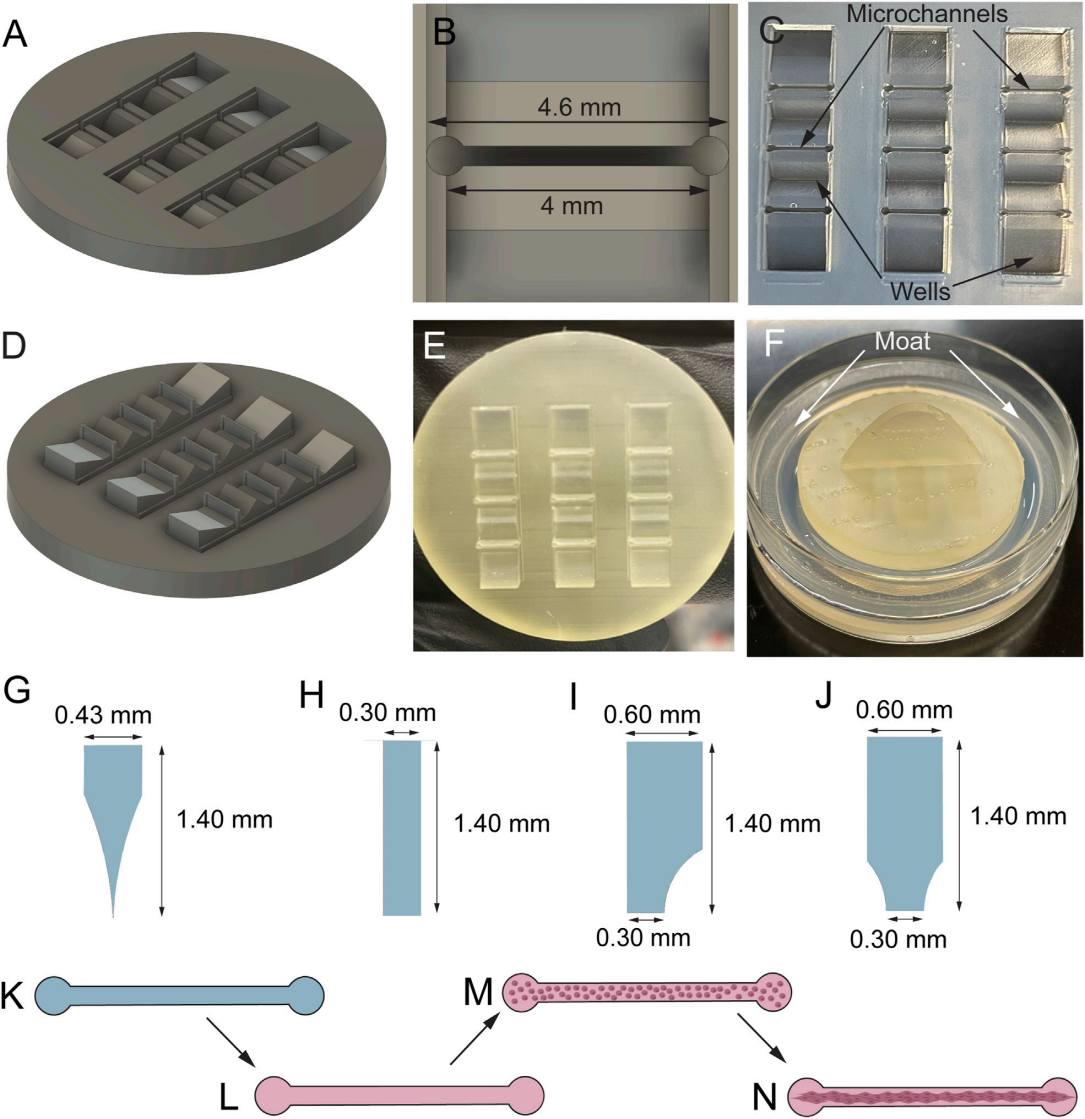
Fabrication of hydrogel microchannels. The stamps were designed in AutoDesk Fusion 360. An example schematic of the final channel grid design **(A)** and a single channel **(B)** are shown. The final product is a 3 × 3 grid of channels in agarose **(C)**. Stamps for 4 different channel shapes (separate stamp for each shape) were printed **(D)** in high-temperature resin **(E)**, allowing them to be autoclaved before each use. The stamps were stamped into hot agarose **(F)** to create a grid of channels **(C)** surrounded by wells that collect liquid away from the channels. A media moat **(F)** was also stamped into the agarose using a separate 3D-printed piece (not shown) made from the same resin. Four different channel shapes–curved triangular **(G)**, rectangular **(H)**, 1-curve rectangular **(I)**, and 2-curve rectangular **(J)** – were tested to investigate which would result in the greatest quantity of robust TE-RMSs. TE-RMSs were fabricated in agarose microchannels **(K)**. Channels were first loaded with collagen **(L)**. Following complete collagen polymerization and drying, a dense cell suspension (2.5 million cells/mL) was loaded into the microchannels **(M)**. Astrocytes self-assemble into longitudinally aligned bundles within a period of 4 days **(N)**.

Hydrogel microchannels were fabricated by stamping a custom stamp into hot agarose (Figure 2). First, a schematic of the final product was created in Autodesk Fusion 360 (Figure 2A). The design contains 9 channels (Figure 2B) that are arranged in a 3-by-3 grid with wells that were designed to collect media away from the channels (Figure 2C). The final design was then reversed to create printable stamps (Figures 2D,E) that would create the desired channel design when stamped into agarose (Figure 2F). Four different channel shapes were designed (Figures 2G–J), with a different stamp designed for each channel shape. All 9 channels within a single stamp were identical. An annulus was also designed and printed that, when stamped into agarose, would create a media moat around the grid of channels (Figure 2F). Stamps were printed in high-temperature resin at the University of Pennsylvania Libraries’ Holman Biotech Commons. Following printing, annuli and stamps were carefully removed from their supports, then cleansed with de-ionized water, wrapped in aluminum foil, and autoclaved. The autoclaved annuli and stamps were brought into a laminar flow hood and removed from the foil. Agarose (Sigma #A9539-500G) and phosphate buffered saline (Gibco #14190136) were added to a beaker in amounts appropriate to make a 3% weight by volume agarose solution. Around 7.5 mL of agarose solution was required for each mold. While the beaker was covered with foil, the contents were heated on a hot plate stirrer at about 200 rpm and above 100°C. Once the solution went from opaque to translucent, the temperature was turned to slightly below 100°C to prevent the agarose from boiling over. The agarose was ready to use once it was completely transparent and devoid of bubbles. 2 mL of agarose were pipetted into a 60 mm sterile dish and spread evenly over the bottom of the dish. The agarose was left to harden for 2–3 min. Then, 5.5 mL of agarose were added on top of the first layer and spread evenly. Immediately thereafter, the annulus was placed into the dish, followed by the stamp (Figure 2F, annulus creating the moat not shown). The agarose was left to harden for 9–10 min. Once the agarose had hardened, the stamp and annulus were removed from the agarose. 3 mL of PBS were added to the dish to keep the mold hydrated. This process was repeated to create the desired number of molds.

### Fabrication of tissue-engineered rostral migratory streams

Microchannel molds were placed under a UV light for sterilization prior to TE-RMS fabrication. PBS was removed from molds with a glass Pasteur pipette and vacuum. Molds were dried several times to ensure no PBS was left inside the channels. Channels (Figure 2K) were seeded with cold (on ice) rat tail type 1 collagen (Advanced BioMatrix #5153) diluted in neutralization buffer containing 50% TE-RMS culture media [a base of Neurobasal media supplemented with 2% B27 (Invitrogen #12587010), 0.25% L-glutamine (Gibco #35050061), 1% G5 supplement (Gibco #17503012), and 1% Penicillin-Streptomycin (Gibco #15140122)], 14.1% cell culture water (Corning #25055CM), 0.5X minimum essential media (Gibco #11430030), 25 mM HEPES (Gibco #15630080), 26.2 mM NaHCO3 (Gibco #25080094), and 0.5X G5 supplement (Gibco #17503012) (Figure 2L). Dishes were placed in an incubator at 37°C and 5% CO_2_ for around 2 h until the collagen completely polymerized to coat the inner walls of the microchannels. During this collagen polymerization period, a flask of 80% confluent astrocytes was passaged with 0.25% trypsin-EDTA (Gibco #25200056) and cells were re-suspended in TE-RMS culture media at a density of 2.5 million cells/mL. Following complete collagen polymerization in the microchannels, astrocytes were seeded into the channels (Figure 2M). 0.75 mL of TE-RMS culture media was added to the moat surrounding the channel grid to act as a humidity chamber and dishes were returned to incubate at 37°C and 5% CO_2_. 1 h later, dishes were flooded with 2.25 mL of TE-RMS culture media and returned to incubate at 37°C and 5% CO_2_. Under these conditions, astrocytes bundled together with collagen to self-assemble into the TE-RMS (Figure 2N). TE-RMSs at DIV4-5 after plating were used for all migration assays. TE-RMSs at DIV7 after plating were used for channel shape comparison assays. TE-RMSs at DIV5 after plating were used for high magnification fluorescent imaging.

### *In vitro* migration assay

SVZ neurospheres at DIV7-8 following isolation and TE-RMSs at DIV4-5 following fabrication (in rectangular channels) were utilized for all *in vitro* migration assays. Only bunded TE-RMSs with ideal morphology (single continuous bundle with no breaks, offshoots, or discontinuous portions) were utilized for these studies. The majority of media was removed from TE-RMS channels and SVZ neurospheres were carefully transferred from 96-well plates into channels. Neurospheres were carefully placed on top of TE-RMSs. Dishes were returned to incubate at 37°C and 5% CO_2_. After 15 min, media was again added to channels and dishes were again returned to incubation conditions. Three different types of culture media were used for this assay (Supplementary Table S1). Half of the media was replaced every 3–4 days. Dishes were live imaged on a confocal microscope at 1 h, days co-cultured (DCC) 2-3, DCC7, DCC9-10, and DCC14 following addition of SVZ cells to TE-RMSs to track the leading edge of EGFP fluorescent signal. Channels were fixed and stained at DCC14 following SVZ cell loading.

### Immunocytochemistry

Cultures were fixed with 4% paraformaldehyde for 30 min at room temperature. For low magnification imaging, TE-RMSs were fixed and stained directly in the microchannels. For high magnification imaging, glass coverslips were coated with 0.002% poly-l-lysine (Sigma #P4707), incubated for 2 h at 37°C, rinsed three times with cell culture water (Corning #25055CM), and left to dry. A small amount of freshly made collagen (see above section on fabrication of tissue-engineered rostral migratory streams and planar astrocyte cultures) was placed on coverslips and a TE-RMS was carefully extracted from channels and laid onto the coverslips in the collagen. Collagen was allowed to dry for 2–3 min prior to fixation. Following rinsing with PBS, cultures were permeabilized with 0.3% triton X-100 and blocked with 4% normal horse serum for 1 hour at room temperature. Following rinsing with PBS, cultures were incubated with primary antibodies overnight at 4°C. Cultures were then incubated in appropriate Alexa secondary antibodies in the dark for 2 hours at room temperature. Following secondary staining, cultures were rinsed with PBS and stored in PBS at 4°C. The TE-RMS that was extracted onto a glass coverslip was rinsed in PBS, rinsed once with deionized water, then mounted onto a glass slide with fluoromount G (Southern Biotech #0100-01). The edges of the glass slide and coverslip was sealed with nail polish and allowed to dry in the dark prior to being stored at 4°C. TE-RMSs were incubated in goat anti-glial fibrillary acidic protein (GFAP) (1:1000) (Abcam #ab53554, RRID: AB_880202) followed by secondary antibody donkey anti-goat 568 (1:500) (Thermo Fisher Scientific #A-11057, RRID: AB_2534104) and Hoechst solution (1:1000) (Thermo Fisher Scientific #H3570). SVZ cells were incubated in goat anti-GFAP (1:1000) (Abcam #ab53554, RRID: AB_880202), mouse anti-nestin (1:100) (Thermo Fisher Scientific #14-5843-82, RRID: AB_1907435), rabbit anti-PAX6 (1:100) (Thermo Fisher Scientific #42-6600, RRID: AB_2533534), rabbit anti-SOX2 (1:100) (Thermo Fisher Scientific #48-1400, RRID: AB_2533841), chicken anti-microtubule associated protein 2 (MAP2) (1:1000) (Abcam #ab5392, RRID: AB_2138153), and rabbit anti-myelin basic protein (MBP) (1:200) (Sigma-Aldrich #M3821, RRID: AB_1841021) followed by secondary staining in donkey anti-rabbit 568 (1:500) (Thermo Fisher Scientific #A10042, RRID: AB_2534017), donkey anti-mouse 647 (1:500) (Thermo Fisher Scientific #A31571, RRID: AB_162542), donkey anti-chicken 647 (1:500) (Jackson ImmunoResearch Labs #703-605-155, RRID: AB_2340379), donkey anti-goat 647 (1:500) (Thermo Fisher Scientific #A-21447, RRID: AB_2535864), and Hoechst solution (1:1000) (Thermo Fisher Scientific #H3570).

For the viability/cytotoxicity assay, media was removed from channels and replaced with a solution of calcein AM (4 μM) and ethidium homodimer-1 (2 μM) diluted in PBS. Cultures were incubated at 37°C and 5% CO_2_ for 30 min, rinsed three times with PBS, then immediately imaged.

### Imaging

Phase images were obtained using a Nikon Inverted Eclipse Ti-S microscope with digital image acquisition using a QiClick camera interfaced with Nikon Elements Basic Research software (4.10.01). Fluorescent images were obtained using a Nikon A1Rsi Laser Scanning Confocal microscope with a ×10, ×20, or ×100 objective (CFI Plan Apo Lambda ×10, n.a. 0.45; ×20, n.a. 0.75; ×100 Oil, n.a. 1.45).

### Imaging analyses, statistics, and reproducibility

Image analyses were performed using FIJI (Fiji is Just ImageJ) software (Schindelin et al., 2012). Statistical testing was performed using GraphPad Prism 10 for macOS. 10× tiled phase images and 10× tiled fluorescent confocal images were utilized for analysis of TE-RMS structure across 4 channel shapes. TE-RMS structure was analyzed by a researcher who was blinded to the shape of the channel that each TE-RMS was fabricated in. First, TE-RMSs were categorized as bundled, not bundled, or collapsed. Microtissues were categorized as bundled if at least 75% of the total microtissue was bundled. Microtissues were categorized as not bundled if less than 75% of the total microtissue was bundled. Microtissues were categorized as collapsed if the TE-RMS was bundled and at least one end of the TE-RMS had detached from the edge of the channel, balled up, and collapsed beyond the circular ends of the channel. Collapsed microtissues only underwent phase microscopy, not fluorescence microscopy. Microtissues that were categorized as bundled then underwent a second round of analysis to examine the structure of the bundles in more detail. Bundles were analyzed for the presence of multiple bundles, off shoots, or discontinuous bundles. Microtissues that had none of these features were categorized as optimal morphology. Microtissues that had one or more of these features were categorized as sub-optimal morphology. Multiple bundles were defined as the presence of two or more distinct bundles within the channel. Off shoots were defined as GFAP processes that were oriented in a different direction than the direction of the main bundle. Discontinuous bundles were defined as TE-RMSs that contained tightly bundled regions interspersed with regions that were not bundled.

10× tiled fluorescent confocal images were utilized for analysis of SVZ-derived cell migration along TE-RMSs. Migration was quantified by tracking the leading edge of EGFP signal from transgenic SVZ cells at each time point. First, images from DCC0, DCC2-3, DCC7, DCC9-10, and DCC14 were aligned such that the SVZ neurosphere starting position was at the same point in each image. An ROI was created at the DCC0 image at the point on the SVZ neurosphere(s) that was protruding the furthest into the channel (i.e., the point that was furthest to the right). This ROI was applied to the DCC2-3, DCC7, DCC9-10, and DCC14 images. A line was drawn from the ROI to the furthest point of EGFP signal visible in each image. The micron length of each line was measured to acquire the distance that SVZ cells had migrated at each time point. Distance migrated at DCC2-3 and DCC14 was compared by ordinary one-way ANOVA and Sidak’s multiple comparisons test. Distance migrated at DCC9-10 and DCC14 was also compared by ordinary one-way ANOVA and Sidak’s multiple comparisons test. These comparisons were performed for 3 different types of culture media utilized in the experiment. Furthest distance migrated at DCC14 across all 3 media types was compared by ordinary one-way ANOVA and Sidak’s multiple comparisons test.

## Results

### Rectangular channel shape produced the most robust and high-throughput TE-RMS

**FIGURE 3:**
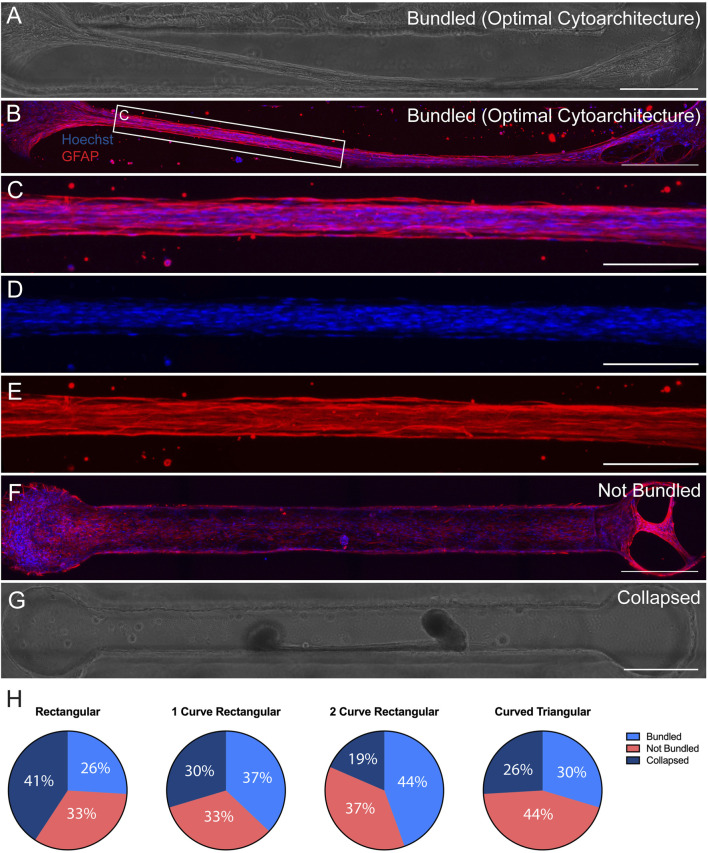
Comparison of TE-RMSs fabricated in 4 channel shapes at DIV7. Astrocyte behavior in channels of 4 different dimesons (*n* = 27 per group) was examined at DIV7 after TE-RMS plating. Example phase contrast image **(A)** and corresponding fluorescent image **(B)** depicting bundled TE-RMS in a 1-curve rectangular channel. Magnified fluorescent view **(C)** depicting bundled TE-RMS with nuclei (Hoechst) in blue **(D)** and aligned astrocyte processes (GFAP) in red **(E)**. Example fluorescent image of not bundled TE-RMS in a rectangular channel with nuclei (Hoechst) in blue and astrocyte processes (GFAP) in red **(F)**. Example phase contrast image of collapsed TE-RMS in a curved triangular channel **(G)**. Pie charts depicting total percentages of bundled, not bundled, and collapsed TE-RMSs in rectangular, 1-curve rectangular, 2-curve rectangular, and curved triangular shapes **(H)**. Scale bars: 500 microns **(A,B and F,G)**, 200 microns **(C–E)**.

**FIGURE 4:**
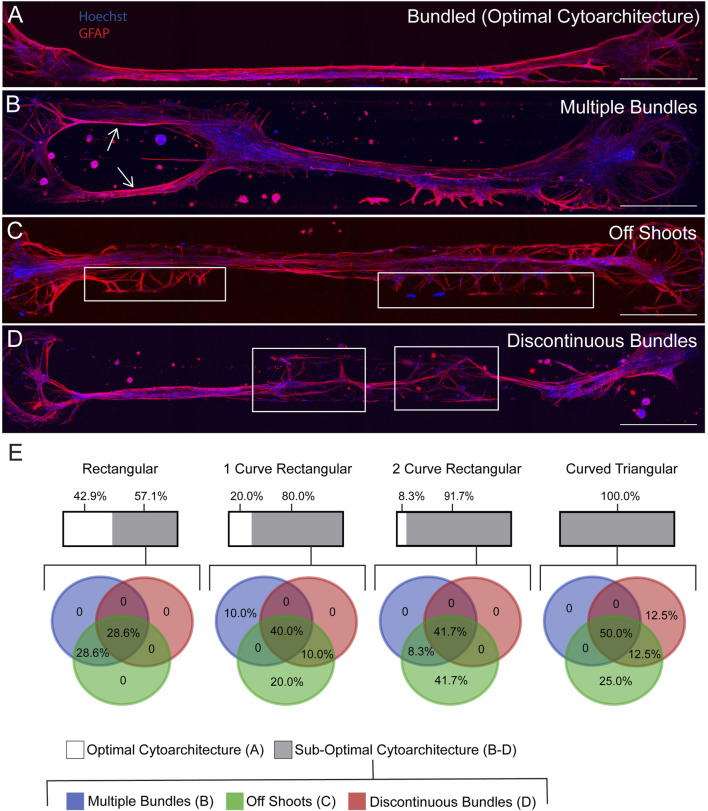
Analysis of multiple bundles, off shoots, and discontinuous bundles in bundled TE-RMSs at DIV7. TE-RMSs that were categorized as bundled (*n* = 7 rectangular, *n* = 10 1-curve rectangular, *n* = 12 2-curve rectangular, *n* = 8 curved triangular) underwent further analysis to examine the structure of the bundles in more detail. Example TE-RMS bundled in a 1-curve rectangular channel **(A)**. Example TE-RMS in a 2-curve rectangular channel with arrows indicating the presence of multiple bundles **(B)**. Example TE-RMS in a rectangular channel with boxed regions indicating the presence of off shoots from the main TE-RMS bundle **(C)**. Example TE-RMS in a curved triangular channel with boxed regions indicating discontinuity in the TE-RMS bundle **(D)**. Graphical representation of results **(E)**. For each channel shape, the upper bar depicts the percentage of bundled microtissues that had optimal morphology in white and sub-optimal morphology in grey. For microtissues that had sub-optimal morphology, an overlapping pie chart shows the specific percentage breakdown of the frequency that each sub-optimal behavior was observed, with multiple bundles depicted in blue, off shoots in green, and discontinuous bundles in red. Pie charts overlap to depict percentage of microtissues that displayed more than one sub-optimal behavior. For all fluorescent images, nuclei (Hoechst) are in blue and astrocytes processes (GFAP) are in red. Scale bars: 500 microns **(A–D)**.

We have previously reported fabrication of the TE-RMS inside hydrogel microcolumns, demonstrating TE-RMS self-assembly inside of a cylindrical tube. Here, we show that the TE-RMS can also self-assemble inside hydrogel microchannels of different dimensions. We observed and imaged astrocyte behavior inside microchannels every other day for 7 DIV. The success of TE-RMS self-assembly inside 4 different dimensions of microchannels–rectangular, 1-curve rectangular, 2-curve rectangular, and curved triangular (Figure 2G)–was compared at the DIV7 time point following TE-RMS fabrication. First, TE-RMSs were placed into three categories based on whether microtissues had bundled, not bundled, or collapsed (Figure 3). Microtissues that were categorized as having bundled into the optimal cytoarchitecture displayed tight bundles of longitudinally aligned astrocytes throughout the channels (Figures 3A–E). Astrocytes in bundled microtissues contained elongated nuclei (Figure 3D) and processes oriented parallel to the direction of the long nuclear axes (Figure 3E). Microtissues that were categorized as not bundled displayed astrocytes spread throughout the channel that had not self-assembled into tight bundles of longitudinally aligned astrocytes (Figure 3F). Microtissues that were categorized as collapsed contained astrocytes that had formed tight bundles of longitudinally aligned astrocytes, but then the contraction of cells that formed the bundle was so high that one or both ends of the bundle collapsed from the edges of the channel (Figure 3G). Pie charts were created to represent the percentage of microtissues that had bundled, not bundled, and collapsed across the 4 channel shapes (*n* = 27 per channel shape) (Figure 3H). In the rectangular channel shape, 26% of the microtissues bundled (*n* = 7), 33% of the microtissues did not bundle (*n* = 9), and 41% of the microtissues collapsed (n = 11). In the 1-curve rectangular shape, 37% of the microtissues bundled (*n* = 10), 33% of the microtissues did not bundle (*n* = 9), and 30% of the microtissues collapsed (*n* = 8). In the 2-curve rectangular channel shape, 44% of the microtissues bundled (*n* = 12), 37% of the microtissues did not bundle (*n* = 10), and 19% of the microtissues collapsed (*n* = 5). In the curved triangular channel shape, 30% of the microtissues bundled (*n* = 8), 44% of the microtissues did not bundle (*n* = 12), and 26% of the microtissues collapsed (*n* = 7). All microtissues that were categorized as bundled (*n* = 7 rectangular, *n* = 10 1-curve rectangular, *n* = 12 2-curve rectangular, *n* = 8 curved triangular) underwent further analysis to examine the structure of the bundles for the presence of multiple bundles, off shoots, or discontinuous bundles (Figure 4). Microtissues that did exhibit any of these cell behaviors were categorized has having optimal cytoarchitecture (Figure 4A). Miicrotissues that had two or more distinct bundles visible within the channel were categorized as having multiple bundles (Figure 4B). Microtissues that had intermediate filament processes oriented in a different direction than the direction of the main bundle were categorized as having off shoots (Figure 4C). Microtissues that had regions of astrocytes that were tightly bundled interspersed with regions of astrocytes that were not bundled were categorized as discontinuous bundles (Figure 4D). Multiple bundles, off shoots, and discontinuous bundles were all categorized as sub-optimal bundling behavior. Some TE-RMSs contained between 1-3 sub-optimal behaviors. Astrocyte characteristics in bundled microtissues were summarized with a combination of bar graphs and overlapping pie charts (Figure 4E). For the rectangular channel shape, 42.9% of bundled microtissues had optimal cytoarchitecture (*n* = 3) and 57.1% had sub-optimal cytoarchitecture (*n* = 4). Of those that were sub-optimal, 28.6% had multiple bundles and off-shoots (*n* = 2) and 28.6% had multiple bundles, off shoots, and were discontinuous (*n* = 2). For the 1-curve rectangular channel shape, 20.0% of the microtissues had optimal cytoarchitecture (*n* = 2) and 80.0% had sub-optimal cytoarchitecture (*n* = 8). Of those that were sub-optimal, 10.0% had multiple bundles (*n* = 1), 20.0% had off shoots (*n* = 2), 10% had off shoots and discontinuous bundles (*n* = 1), and 40% had multiple bundles, off shoots, and discontinuous bundles (*n* = 4). For the 2-curve rectangular channel shape, 8.3% of microtissues had optimal morphology (*n* = 1) and 91.7% of microtissues had sub-optimal morphology (*n* = 11). Of those that were sub-optimal, 41.7% had off shoots (*n* = 5), 8.3% had multiple bundles and off shoots (*n* = 1), and 41.7% had multiple bundles, off shoots, and discontinuous bundles (*n* = 5). For the curved triangular shape, 100.0% of microtissues had sub-optimal morphology. 25.0% had off shoots (*n* = 2), 12.5% had discontinuous bundles (*n* = 1), 12.5% had off shoots and discontinuous bundles (*n* = 1), and 50% had multiple bundles, off shoots, and discontinuous bundles (*n* = 4). The rectangular channel shape was chosen for the rest of experiments herein because it produced the greatest quantity of TE-RMSs with bundled, optimal morphology. Here, we compared TE-RMS behavior at DIV7 following plating. However, during these experiments we observed that TE-RMSs formed bundled, optimal morphology by DIV4 following plating. Thus, TE-RMSs at DIV4 were utilized for the rest of the experiments herein.

### High magnification fluorescent imaging highlights that TE-RMSs fabricated in rectangular channels have aligned, bidirectional processes and elongated nuclei

**FIGURE 5:**
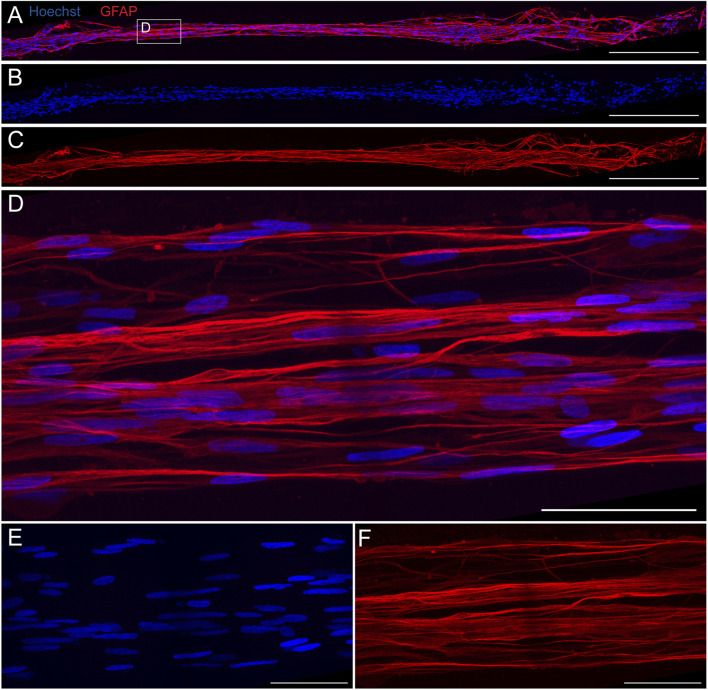
High magnification fluorescent imaging reveals structure of TE-RMS fabricated in rectangular channel. TE-RMS microtissue extracted from rectangular channel at DIV4 at 20× **(A–C)** depicting merged channels **(A)**, nuclei Hoechst, **(B)**, and astrocyte processes GFAP, **(C)**. Magnified ×100 view **(D–F)** clearly depicts elongated nuclei **(E)** and aligned, bidirectional processes **(F)** of TE-RMS astrocytes. Scale bars: 500 microns **(A–C)** and 50 microns **(D–F)**.

Our previous work demonstrated that TE-RMSs fabricated in hydrogel microcolumns have elongated nuclei and cytoskeletal rearrangement consistent with the endogenous RMS (Purvis et al., 2022) (Figure 1). Here, a TE-RMS at DIV4 was extracted from a rectangular shaped microchannel and high magnification (×100) fluorescent confocal imaging was used to examine the microstructure in more detail (Figures 5A–F). A 20× confocal image shows the length of the entire TE-RMS (Figure 5A), highlighting nuclear (Figure 5B) and intermediate filament (Figure 5C) channels. A magnified ×100 view (Figures 5D–F) shows detailed configuration of cells within the TE-RMS. TE-RMS astrocytes possess elongated nuclei (Figure 5E) and intermediate filaments extending in two directions parallel to the direction of the long nuclear axis (Figure 5F). Thus, TE-RMSs fabricated in hydrogel microchannels exhibit the same unique architecture of TE-RMSs fabricated in hydrogel microcolumns.

### Neurospheres harvested from the rat subventricular zone express neural precursor cell markers

To demonstrate feasibility of harvesting and culturing SVZ tissue, SVZ tissue was harvested from 3 different rat strains that constitutively express EGFP [SD-Tg(UBC-EGFP)2BalRrrc (heterozygous), LEW-Tg(CAG-EGFP)YsRrrc (hemizygous), F344-Tg(UBC-EGFP)F455Rrrc (homozygous)] (*n* = 2 subjects per species). Cells from all 3 rat strains demonstrated robust EGFP signal and matured into both astrocytes and neurons *in vitro* (data not shown). Strain SD-Tg(UBC-EGFP)2BalRrrc (heterozygous) was utilized for all future studies so that all components of the *in vitro* culture system were isolated from Sprague-Dawley rats.

**FIGURE 6:**
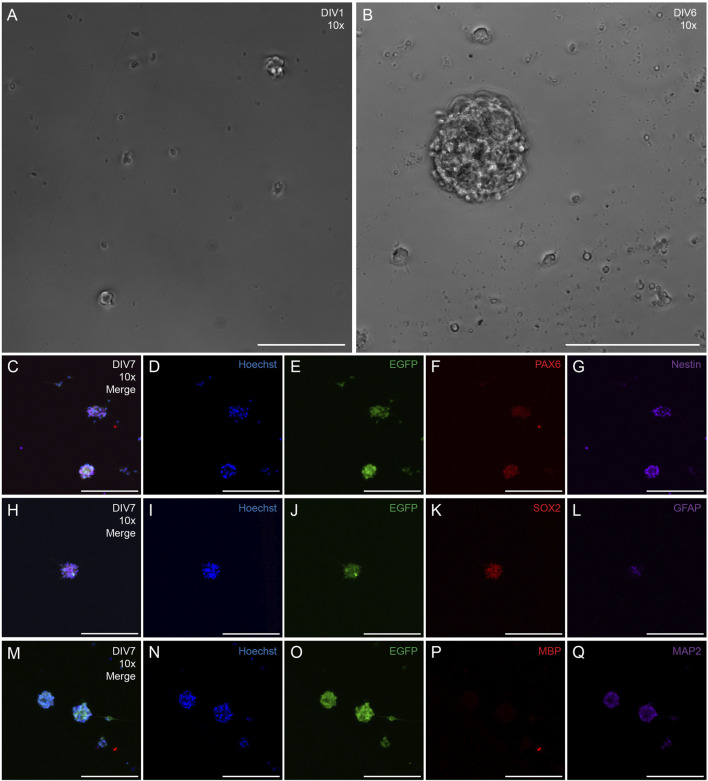
Phenotypic characterization of neurospheres derived from the SVZ of transgenic adult rats that ubiquitously express EGFP. Representative 10x phase images of SVZ neurospheres in culture at DIV1 **(A)** and DIV6 **(B)** following harvest, Representative 10x fluorescent image of DIV7 neurosphere with merged channels **(C)**, nuclei (Hoechst, **(D)**, EGFP **(E)**, and neural progenitor cell markers PAX6 **(F)** and nestin **(G)**. Representative 10x fluorescent image of DIV7 neurosphere with merge channels **(H)**, nuclei (Hoechst, **(I)**, EGFP **(J)**, and neural progenitor cell markers SOX2 **(K)** and GFAP **(L)**. Representative 10x fluorescent image of DIV7 neurosphere with merged channels **(M)**, nuclei [(Hoechst **(N)**], EGFP **(O)**, mature oligodendrocyte marker myelin basic protein [MBP **(P)**], and mature neuronal marker microtubule-associated marker 2 [MAP2 **(Q)**]. Scale bars: 200 microns **(A–Q)**.

Neural precursor cells were isolated from the SVZ of rat strain SD-Tg(UBC-EGFP)2BalRrrc (heterozygous) and cultured as neurospheres for 10 DIV (Figure 6), Neurospheres were visualized under phase microscopy at DIV1 (Figure 6A) and DIV6 (Figure 6B). Phenotypic characterization was examined at DIV7-10 after isolation of the cells. SVZ-derived cells stained positive for nuclei (Hoechst; Figures 6D,I, N), EGFP (Figures 6E,J,O), and active neural stem cell markers PAX6 (Figure 6F), Nestin (Figure 6G), SOX2 (Figure 6K), and GFAP (Figure 6L). *N* = 36 neurospheres were analyzed for PAX6 and nestin expression. *N* = 45 neurospheres were analyzed for SOX2 expression. 100% of analyzed neurospheres stained positive for PAX6 (*n* = 36), nestin (*n* = 36), and SOX2 (*n* = 45). *N* = 45 neurospheres were analyzed for GFAP expression. 69% of analyzed neurospheres had some GFAP expression (*n* = 31) whereas 31% of analyzed neurospheres did not stain positive for GFAP (*n* = 14). *N* = 33 neurospheres were analyzed for mature oligodendrocyte marker MBP and mature neuronal marker MAP2. 100% of analyzed neurospheres showed negative expression of MBP (*n* = 33) (Figure 6P) and some marker positivity (limited expression) of MAP2 (*n* = 33) (Figure 6Q).

### Rat subventricular zone-derived cells migrate through the TE-RMS *in vitro*

**FIGURE 7:**
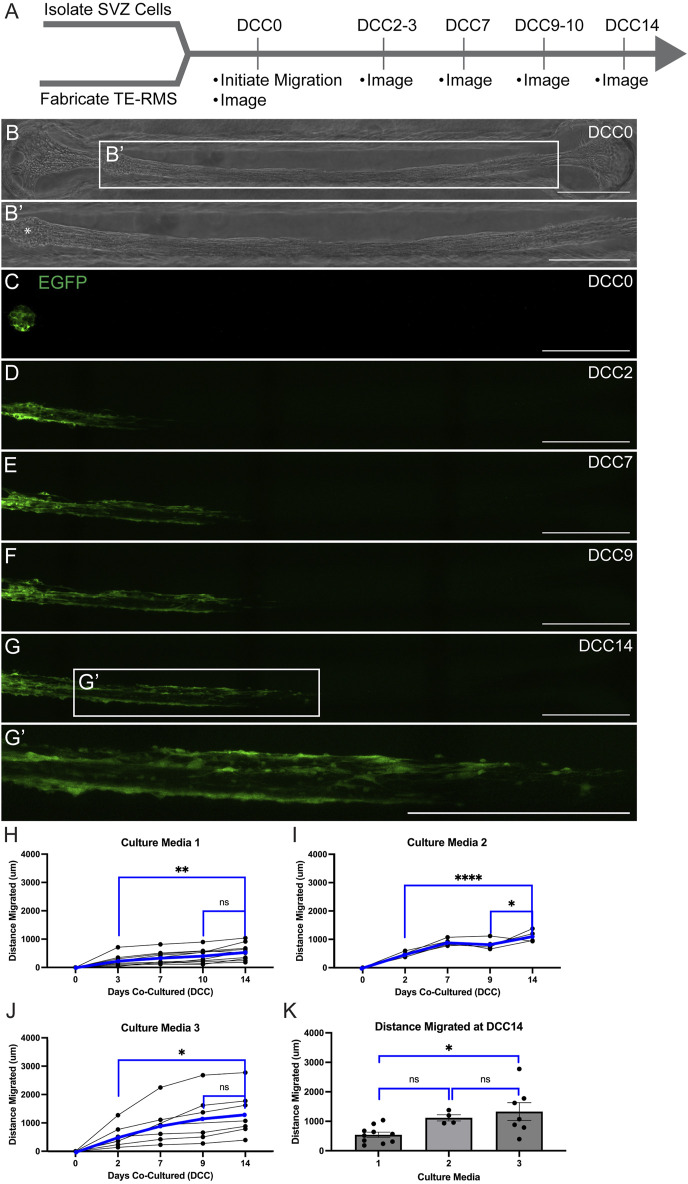
Live migration of SVZ-derived EGFP+ cells through TE-RMS. Experimental timeline **(A)**. SVZ cells were DIV7 and TE-RMSs were DIV4 when migration was initiated. Phase image of TE-RMS in a rectangular channel 1 h after loading of SVZ neurosphere into the channel [DCC0; **(B)**]. Magnified view depicts the structure of the TE-RMS in more detail **(B’)**. Star (*) indicates where the neurosphere landed upon loading **(B’)**. Fluorescent imaging depicting live migration of SVZ-derived EGFP+ cells through the TE-RMS **(C–G’)** 1 h after loading SVZ cells [DCC0, **(C)**], DCC2 after loading **(D)**, DCC7 after loading **(E)**, DCC9 after loading **(F)**, and DCC14 after loading **(G)**. Magnified view depicts multiple chains of EGFP+ cells migrating through the TE-RMS at DCC14 **(G’)**. Migration was tracked by measuring the leading edge of EGFP signal in three different types of culture media **(H–K)**. One-way ANOVAs with Sidak’s *post hoc* multiple comparison tests were used to compare distance migrated at DCC2-3 *versus* DCC14 and at DCC9-10 *versus* DCC14 **(H–J)**. In each graph, black lines represent individual samples and blue lines represent the average **(H–J)**. One-way ANOVA with Sidak’s *post hoc* multiple comparison test was used to compare furthest distance migrated at DCC14 between 3 media types **(K)**. Scale bars: 500 microns **(B–G’)**. *p < 0.05; **p < 0.01; ****p < 0.0001.

We loaded SVZ-derived neurospheres on top of one end of fully formed TE-RMSs and examined the migration of SVZ cells along the TE-RMS for 14 DCC (Figure 7). SVZ neurospheres were DIV7 at the start of the assay and TE-RMSs were DIV4 at the start of the assay. An example representative phase image (Figure 7B) with magnified view (Figure 7B’) shows the structure of the TE-RMS bundle at 1 h after SVZ-derived neurospheres were loaded onto the channel (DCC0). The star indicates where the neurosphere landed upon loading (Figure 7B’). Live migration of SVZ-derived neurospheres was tracked with repeated measures confocal imaging at 1 h (DCC0; Figure 7C), DCC2-3 (Figure 7D), DCC7 (Figure 7E), DCC9-10 (Figure 7F, and DCC14 (Figure 7G) following SVZ cell loading. The magnified view at DCC14 (Figure 7G’) depicts multiple chains of EGFP+ cells migrating through the TE-RMS. In trying to design the most optimal platform, migration was assessed in 3 different types of culture media. Media constituents are depicted in Supplementary Table S1. Migration of EGFP+ cells was assessed by measuring the furthest distance of EGFP signal at each time point. Data is graphically depicted with each line representing a different sample (Figures 7H–J). In each media type, the distance migrated at DCC2-3 and DCC14 and the distance migrated at DCC9-10 and DCC14 were directly compared by one-way ANOVAs with Sidak’s *post hoc* multiple comparison tests. In each graph, black lines represent individual samples and blue lines represent the average. In culture media 1 (*n* = 10), SVZ-derived cells had migrated significantly further by DCC14 compared to DCC3 (*p* = 0.0050) (Figure 7H). Distance migrated in culture media 1 was not significantly different between DCC 14 and DCC10 (*p* = 0.3328) (Figure 7H). In culture media 2 (*n* = 5), SVZ-derived cells had migrated significantly further by DCC14 compared to DCC2 (*p* < 0.0001) (Figure 7I). Cells in culture media 2 also migrated significantly further by DCC14 compared to DCC9 (*p* = 0.0347) (Figure 7I). Like SVZ cells in culture medias 1 and 2, SVZ-derived cells in culture media 3 (*n* = 7) had migrated significantly further by DCC14 compared to DCC 2 (*p* = 0.0486) (Figure 7J). There was no significant difference in migration distance between DCC14 and DCC9 in culture media 3 (*p* = 0.9017) (Figure 7J). The average distance migrated by DCC14 between the three types of media was compared by one-way ANOVA with Sidak’s *post hoc* multiple comparison test. SVZ-derived cells in media 3 had migrated significantly further by DCC14 compared to SVZ cells in media 1, whereas there was no difference in migration distance between cells in media 1 *versus* 2 or media 2 *versus* 3 (Figure 7K).

**FIGURE 8:**
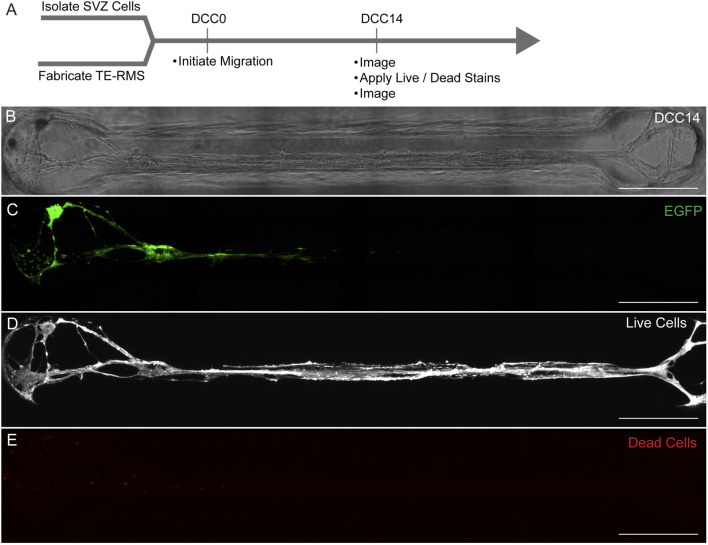
TE-RMS viability at DCC14. Experimental timeline **(A)**. Phase image of TE-RMS in a rectangular channel 14 days after loading SVZ cells to the end of the TE-RMS **(B)**. Live migration of SVZ cells depicted with EGFP signal at DCC14 **(C)**. Live cells visualized with calcein AM **(D)** and dead cells visualized with ethidium homodimer-1 **(E)**. Quantity of live cells is estimated to be >95%. Scale bars: 500 microns **(B–E)**.

Cell viability was assessed at DCC14 following loading of SVZ-derived cells into the TE-RMS (*n* = 5) (Figure 8). A representative microtissue is depicted here with a phase image (Figure 8B) and a fluorescent image of live SVZ-derived cell migration taken right before viability/cytotoxicity staining was applied (Figure 8C). Images depicting live cells (Figure 8D) and dead cells (Figure 8E) reveal that most cells are alive at DCC14 following SVZ neurosphere loading. We estimate the quantity of live cells to be >95%.

SVZ-derived neurospheres isolated from LEW-Tg(CAG-EGFP)YsRrrc (hemizygous) and F344-Tg(UBC-EGFP)F455Rrrc (homozygous) rat strains were also loaded onto the end of fully-formed TE-RMSs (Supplementary Figure S1). Microtissues were live imaged 1 h (DCC0; Supplementary Figures S1B,G) following SVZ-derived cell loading. Microtissues were fixed at DCC5 after SVZ cell loading and stained with GFAP and Hoechst (Supplementary Figures S1C,H–K). Images depict live migration of SVZ-derived cells (Supplementary Figures S1D,I) along longitudinally aligned astrocytes (Supplementary Figures S1F,K). These data collectively indicate that SVZ-derived cells from 3 strains of EGFP+ rats [SD-Tg(UBC-EGFP)2BalRrrc, LEW-Tg(CAG-EGFP)YsRrrc, F344-Tg(UBC-EGFP)F455Rrrc] can migrate along the TE-RMS *in vitro*.

## Discussion

The TE-RMS is the first biomimetic tissue-engineered technology that is designed for stable and sustained neuronal replacement *in vitro* and *in vivo*. In addition to the potential of the TE-RMS as a regenerative medicine strategy for endogenous neuronal replacement into a neuron-deficient brain region following injury, the TE-RMS also serves as an *in vitro* tool to unlock mechanisms of neuronal migration in more detail. Our previous *in vivo* and *in vitro* TE-RMS studies have used TE-RMSs that were fabricated in hydrogel microcolumns (Winter et al., 2016; Katiyar et al., 2018; O’Donnell et al., 2021; Purvis et al., 2022). TE-RMSs rapidly self-assemble inside microcolumns to form longitudinally aligned bundles of astrocytes interwoven with collagen and tethered to either end of the column (Winter et al., 2016; Katiyar et al., 2018). The curvature of the microcolumn and the density of loaded astrocytes are key mechanical cues for promoting TE-RMS formation (Winter et al., 2016). We have shown that astrocytes of the TE-RMS have elongated nuclei and bidirectional intermediate filament processes that run parallel to the long nuclear axes of the cells–distinct morphology that mimics astrocytes in the endogenous rat RMS (Purvis et al., 2022). In the current report, we demonstrate successful fabrication of TE-RMS microtissues in hydrogel microchannels of different dimensions. We have developed a robust pipeline to design and print 3D reverse molds that are stamped into agarose to create a grid of channels that are then used for TE-RMS fabrication. Fabrication in hydrogel microchannels increased both the speed of the fabrication process and the throughput of TE-RMS microtissues fabricated. With hydrogel microchannels, we fabricated 72 TE-RMS microtissues in 5 h. It would take 18 h to fabricate this same number of TE-RMSs in microcolumns. Additionally, most TE-RMSs fabricated in microcolumns (estimated to be greater than 80%) collapsed within 1 day after fabrication. TE-RMSs fabricated in microchannels collapsed at a much lower rate of 19%–41% (Figure 3H). Developing this higher-throughout microchannel fabrication technique–which allowed us to fabricate more TE-RMSs in a faster time frame–made it possible to examine the *in vitro* applications of the TE-RMS in more detail. Observing astrocyte responses to different shapes of microchannels provided more insight into the specific topographical features that are capable of inducing TE-RMS formation. The 4 shapes of microchannels utilized in this experiment were chosen based on previous experimental observations of astrocyte behavior during TE-RMS fabrication in microcolumns. During previous experiments, we sometimes observed robust TE-RMS formation on the outside of a microcolumn–at the intersection of the convex curved edge of the microcolumn and the straight edge of the bottom of the dish–rather than the inside of the microcolumn where the TE-RMS was supposed to form. Thus, we decided to investigate TE-RMS formation in microchannels composed of various intersections of straight and curved edges. The 1-curved rectangular microchannel shape (Figure 2I) mimicked the topography of the outside of a microcolumn sitting on a straight edge. The 2-curved rectangular microchannel shape (Figure 2J) included this curved feature on both sides of the microchannel. The curved triangular microchannel shape (Figure 2G) mimicked the outer edges of two adjacent microcolumns. The rectangular microchannel shape (Figure 2H) did not contain any curvature. Given the historically low success rates of TE-RMS formation in cylindrical microcolumns, we chose not to include a curved microchannel (mimicking half of a microcolumn) in the current experiments. Interestingly, astrocytes bundled into TE-RMS morphology in all 4 channel shapes utilized in the current study. Thus, TE-RMS formation is possible when astrocytes are topographically exposed to the intersection of 2 straight edges (rectangular and 1-curve rectangular channel shapes), the intersection of 1 straight edge and 1 curved edge (1-curve rectangular and 2-curve rectangular channel shapes), and the intersection of 2 curved edges (curved triangular channel shape). This adds to previous knowledge that TE-RMS formation is possible when astrocytes are exposed to a cylindrical tube (Winter et al., 2016; Katiyar et al., 2018).

When comparing TE-RMS fabrication efficiency across the 4 microchannel shapes, we presumed that straight, non-bifurcated bundles would be the most optimal TE-RMS cytoarchitecture. We assumed that off-shoots or branches from the main bundle could potentially cause cells to migrate in different directions that could lead them astray from their main target end point. Thus, the most direct migration route would likely be the most effective migration pathway. However, it is possible that, in the presence of chemo-attractive factors, cells would migrate directly toward an end target region via the most direct migration route and bypass any branches or off shoots from the most direct path. Future studies will examine neuroblast migration routes in bifurcated TE-RMSs, the impact that microtissue bifurcation has on neuroblast arrival into destination cultures containing various chemo-attractive factors, and the impact of chemo-attractive factors on the choice of migration route. Under the assumption that the most direct migration route is the most optimal, the rectangular channel shape produced the most robust TE-RMSs with the most optimal cytoarchitecture. The convex curvature of the 1-curve rectangular, 2-curve rectangular, and curved triangular channel shapes produced more frequent bifurcations, off shoots, and discontinuities in the TE-RMS compared to the straight walls of the rectangular channel. We speculate that when astrocytes extend to pull polymerized collagen off the edges of convex curvature, that this curvature causes cells to move in various directions more often than when cells pull polymerized collagen off the edges of straight edges. Interestingly, TE-RMS microtissues that were classified as having sub-optimal cytoarchitecture were more likely to have multiple sub-optimal features as opposed to having just one of these features. We observed this pattern across all 4 channel shapes. In previous experiments, TE-RMSs fabricated in microcolumns were sometimes discontinuous and never produced bifurcations or off shoots. Importantly, we see the unique morphological features of TE-RMS astrocytes–elongated nuclei and bidirectional, directionally aligned processes–in microtissues fabricated in microchannels. As we continue studies on the translational therapeutic potential of the TE-RMS as an endogenous neuronal replacement strategy following injury, we are currently working to optimize the TE-RMS fabrication pipeline even further.

In the current study, we demonstrate that we can reliably isolate SVZ tissue and culture SVZ neurospheres from three different strains of rats that constitutively express EGFP [SD-Tg(UBC-EGFP)2BalRrrc, LEW-Tg(CAG-EGFP)YsRrrc, and F344-Tg(UBC-EGFP)F455Rrrc]. SVZ-derived EGFP+ cells from all 3 strains migrated along the TE-RMS *in vitro*. We chose to use cells from strain SD-Tg(UBC-EGFP)2BalRrrc so that all cell components of our *in vitro* system were isolated from Sprague-Dawley rats. We showed that SVZ-derived neurospheres from this rat strain reliably expressed nestin, SOX2, and PAX6, which are markers of neural stem cells (Zhang and Jiao, 2015). Whereas all analyzed neurospheres stained positive for nestin, SOX2, and PAX6 markers, a subset of cells from all neurospheres stained positive for GFAP. This staining pattern is characteristic of active neural stem cells, with GFAP+ signal representing cells that are either astrocytes or type B stem cells (Gil-Perotín et al., 2013). All SVZ-derived neurospheres stained negative for the mature oligodendrocyte marker MBP, whereas a subset of cells in all neurospheres stained positive for the mature neuronal marker MAP2. Thus, our SVZ-derived cells used in the current study might be more prone to mature into a neuronal phenotype under these culture conditions. This evidence collectively demonstrates that we have the tools to reliably isolate, culture, and phenotypically verify the identity of SVZ neurospheres that we used for our migration studies. The EGFP+ cells migrating from SVZ neurospheres and along the TE-RMS are putative neuroblasts, although further experiments are necessary to confirm this. Future experiments will also examine how migration through the TE-RMS affects cell differentiation and how the TE-RMS could be manipulated to control the phenotypic fate of migrating cells.

SVZ neuroblasts migrate in chain formation at a composite rate of between 30 and 70 microns per hour along astrocytes of the endogenous RMS (Lois and Alvarez-Buylla, 1994; Nam et al., 2007; Kojima et al., 2010; Grade et al., 2013; Ota et al., 2014). In the current study, SVZ-derived EGFP+ cells migrated through the TE-RMS at a peak rate of 13 microns per hour over the first 7 days, which is slower than the average rate of migration through the endogenous RMS. We previously reported that aggregated immature rat cortical neurons migrated through the human gingiva mesenchymal stem cell-derived TE-RMS at an average rate of at least 56 microns/hour which falls within the range of the typical migration rate of neuroblasts through the endogenous rat RMS (O’Donnell et al., 2021). In these previous studies, immature rat cortical neurons were aggregated and placed on one end of the TE-RMS. There are several explanations for the slow migration rate that we observed for SVZ-derived EGFP+ cells in the current study. These explanations assume that EGFP+ cells migrating from SVZ neurospheres are neuroblasts. One possibility could be that our *in vitro* system lacks chemo-attractive cues that are found *in vivo*. Various local and remote signaling factors and directional cues, including neurotransmitters and growth factors, guide neuroblasts on their journey through the endogenous RMS toward the OB (Ming and Song, 2011; Christie and Turnley, 2012; Lim and Alvarez-Buylla, 2016; Dillen et al., 2019). Additionally, following brain injury, SVZ neuroblasts can divert from their regular migration route along the RMS and migrate toward injured brain regions (Kojima et al., 2010; Young et al., 2011; Lindvall and Kokaia, 2015; Chang et al., 2016). This migration toward damaged regions is facilitated by chemo-attractants released from injured tissue, including stromal-derived factor 1 (SDF-1 or CXCL12), monocyte chemoattractant protein 1 (MCP1 or CCL2), and osteopontin 1 (Chang et al., 2016; Dillen et al., 2019; Fujioka et al., 2019; Kaneko et al., 2017; Lindvall and Kokaia, 2015; Ohab et al., 2006; Sawada et al., 2014; B; Wang and Jin, 2015). Lack of such chemo-attractive cues in our current *in vitro* system could be responsible for the slow migration rate of SVZ-derived cells (putative neuroblasts) through the TE-RMS. Another explanation could be related to the starting density of neurospheres that were loaded into the system. Due to the technical challenge of precisely loading SVZ neurospheres on the TE-RMS, a low density of cells were loaded onto each TE-RMS for migration studies. The neuronal aggregates used in our previous study were much larger than the SVZ-derived neurospheres loaded onto the TE-RMS in the current studies. SVZ-derived neurospheres were unable to survive the forced aggregation process utilized in previous studies. Thus, we loaded single neurospheres to the end of TE-RMSs to examine their migration rate. This low starting density of SVZ-derived cells, which is far less than the density of neuroblasts both in the SVZ *in vivo* and in our previous TE-RMS migration studies with rat cortical neuronal aggregates, could be related to the speed and distance with which cells migrated from their place of origin. However, our data clearly indicates that SVZ-derived EGFP+ cells are primed to migrate in chain formation along the TE-RMS. Additionally, the high cell viability in our system at 14 days following the initiation of migration studies indicates that cell health is not affecting the slow migration rate. We are currently examining how the controlled addition of chemo-attractive factors to the distal end of the TE-RMS and increased density of neurosphere loading modulate the rate of EGFP+ cell migration along the TE-RMS.

## Conclusion

In this study, we report successful fabrication of TE-RMSs in hydrogel microchannels of 4 different dimensions, with the rectangular channel producing the most robust and high-throughput TE-RMSs. We also provide evidence that cells from isolated SVZ neurospheres from rats that constitutively express EGFP can migrate along TE-RMSs fabricated in rectangular channels *in vitro*. Characteristics of cell-cell communication and neuroblast migration are poorly understood and difficult to study in the brain *in vivo*. Our biomimetic system that replicates a source of migrating SVZ-derived cells along a replicated RMS provides a highly biofidelic platform to investigate mechanisms of cell-cell communication and neuroblast migration *in vitro*. We are currently conducting experiments to verify the phenotype of migrating EGFP+ cells as SVZ neuroblasts. Current experiments are also adding a “destination culture” to the distal end of the TE-RMS, which will allow for examination of the chemical and molecular cues regulating neuroblast maturation, integration, and physiological function after migrating through the TE-RMS. We are also fabricating this system entirely of human cell sources, creating the first system to investigate human neuroblast-astrocyte interactions and neuroblast migration *in vitro*. Current experiments are also examining differential gene expression of TE-RMS astrocytes *versus* planar astrocyte samples, exploring the potential of the TE-RMS as a genetic tool to study the endogenous RMS. Overall, the TE-RMS platform will allow us to uncover pivotal signaling mechanisms that will shed light on molecular cues that can be used to further investigate the process of immature neuronal migration.

## Data Availability

The raw data supporting the conclusions of this article will be made available by the authors, without undue reservation.

## Supplementary material

SUPPLEMENTARY FIGURE S1 SVZ-derived EGFP+ cells harvested from multiple EGFP+ rat strains migrate through the TE-RMS in vitro. Experimental timeline **(A)**. Neural progenitor cells were harvested from the SVZ of strains LEW-Tg(CAG-EGFP)YsRrrc (hemizygous) **(B-F)** and F344-Tg(UBC-EGFP)F455Rrrc (homozygous) **(G-K)**. 7 days following harvest, neurospheres were loaded into one end of DIV4 TE-RMS microtissues. EGFP+ cell migration across TE-RMS is visible when imaged in live culture 1 hour following neurosphere loading [DCC0 (**B, G**)]. TE-RMSs were fixed at DCC5 and labeled with GFAP and Hoechst **(C–F, H–K)**. DCC5 cultures depicting merged channels **(C, H)**, EGFP+ migrating cells **(D, I)**, Hoechst **(E, J)**, and GFAP **(F, K)**. Scale bars: 500 microns **(B–K)**.
