# Supplementary material for: A three-dimensional tissue-engineered rostral migratory stream as an in vitro platform for subventricular zone-derived cell migration
Source: Front Bioeng Biotechnol. 2024 Jun 12;12:1410717. doi: 10.3389/fbioe.2024.1410717 (PMC11199690; doi:10.3389/fbioe.2024.1410717)
Supplement: Supplementary file 1 [file Table1.DOCX]

|  | TE-RMS Culture MEdia | | Neuron-astrocyte co-culture media | SVZ Culture MEdia | Migration Media 1 | Migration  Media 2 | Migration  Media 3 |
| --- | --- | --- | --- | --- | --- | --- | --- |
| Component |  | **CONCENTRATION** | | | | | |
| B27 | 2% | | 2% | 2% | 2% | 2% | 2% |
| L-glutamine | 0.5 mM | | 0.5 mM | 2 mM | 0.5 mM | 2 mM | 2 mM |
| Penicillin Streptomycin | 100 U/mL | | 100 U/mL | 100 U/mL | 100 U/mL | 100 U/mL | 100 U/mL |
| G5 | 1% | | 1% |  | 1% |  |  |
| Glucose |  | |  | 3 mg/mL |  | 3 mg/mL | 3 mg/mL |
| Heparin |  | |  | 2 ug/mL |  | 2 ug/mL | 2 ug/mL |
| Fibroblast growth factor | 5 ng/mL * | | 5 ng/mL * | 20 ng/mL | 5 ng/mL * |  | 20 ng/mL |
| Epidermal growth factor | 10 ng/mL * | | 10 ng/mL * | 20 ng/mL | 10 ng/mL * |  | 20 ng/mL |

Supplementary Table 1. Media constituents and concentrations for TE-RMS culture media, SVZ cell culture media, and SVZ-TE-RMS migration medias. All medias are Neurobasal based. * Refers to EGF and FGF that come from G5 supplement.
